# Identification of Root Rot Resistance QTLs in Pea Using *Fusarium solani* f. sp. *pisi*-Responsive Differentially Expressed Genes

**DOI:** 10.3389/fgene.2021.629267

**Published:** 2021-08-05

**Authors:** Bruce A. Williamson-Benavides, Richard M. Sharpe, Grant Nelson, Eliane T. Bodah, Lyndon D. Porter, Amit Dhingra

**Affiliations:** ^1^Molecular Plant Sciences, Washington State University, Pullman, WA, United States; ^2^Department of Horticulture, Washington State University, Pullman, WA, United States; ^3^USDA-ARS, Grain Legume Genetics and Physiology Research Unit, Prosser, WA, United States

**Keywords:** root rot, quantitative trait loci, SNP, molecular marker, RNAseq, *Pisum sativum* L., disease resistance

## Abstract

*Pisum sativum* (pea) yields in the United States have declined significantly over the last decades, predominantly due to susceptibility to root rot diseases. One of the main causal agents of root rot is the fungus *Fusarium solani* f. sp. *pisi* (*Fsp*), leading to yield losses ranging from 15 to 60%. Determining and subsequently incorporating the genetic basis for resistance in new cultivars offers one of the best solutions to control this pathogen; however, no green-seeded pea cultivars with complete resistance to *Fsp* have been identified. To date, only partial levels of resistance to *Fsp* has been identified among pea genotypes. SNPs mined from *Fsp*-responsive differentially expressed genes (DEGs) identified in a preceding study were utilized to identify QTLs associated with *Fsp* resistance using composite interval mapping in two recombinant inbred line (RIL) populations segregating for partial root rot resistance. A total of 769 DEGs with single nucleotide polymorphisms (SNPs) were identified, and the putative SNPs were evaluated for being polymorphic across four partially resistant and four susceptible *P. sativum* genotypes. The SNPs with validated polymorphisms were used to screen two RIL populations using two phenotypic criteria: root disease severity and plant height. One QTL, *WB.Fsp-Ps* 5.1 that mapped to chromosome 5 explained 14.8% of the variance with a confidence interval of 10.4 cM. The other four QTLs located on chromosomes 2, 3, and 5, explained 5.3–8.1% of the variance. The use of SNPs derived from *Fsp*-responsive DEGs for QTL mapping proved to be an efficient way to identify molecular markers associated with *Fsp* resistance in pea. These QTLs are potential candidates for marker-assisted selection and gene pyramiding to obtain high levels of partial resistance in pea cultivars to combat root rot caused by *Fsp*.

## Introduction

Pea (*Pisum sativum* L.; Family Fabaceae) is an important cool-season, self-pollinating annual diploid crop. A number of cultivars within the species cater to different consumption markets. Green peas, and dry yellow and green peas are sold as food in the fresh and dry markets, respectively, while purple-seeded lines are used for forage and green manure (Miller et al., 2005). Due to its high protein content (20–30%) and overall high nutritional value, pea has become a major contributor to the plant-based protein market (do Carmo et al., 2016; Peng et al., 2016; Xiong et al., 2018; Wei et al., 2020). The shift to plant-based protein is an environmentally sustainable alternative to animal-based protein because the latter contributes significantly to greenhouse gas emissions (Stehfest et al., 2009). Furthermore, studies have shown that dietary proteins in peas are of great benefit to human health and wellness (Reddy and Yang, 2011; Kudre et al., 2013; Dahiya et al., 2015). Consequently, the pea protein market was projected to reach $313.5 million in 2025 (Grand View Research, 2017; Sim et al., 2019).

The profitable production of pea is threatened by soilborne diseases. These diseases are commonly referred to as the pea root rot complex (PRRC) and are caused by a single or combination of pathogens, including *Aphanomyces euteiches*, *Fusarium* spp., *Mycosphaerella pinodes*, *Pythium* spp., and *Rhizoctonia solani* (Xue, 2003; Kumari and Katoch, 2020). One of the predominant causal agents of PRRC is the fungus *Fusarium solani* f. sp. *pisi* (*Fsp*). *Fsp* occurs in most pea fields throughout the world, and the yields of *P. sativum* cultivars can be reduced up to 15–62% by this pathogen (Seaman, 1976; Grünwald et al., 2003). *Fsp* infects pea seeds during germination, with symptoms of root rot beginning at or near the cotyledon-hypocotyl junction and progressing under the soil and upper region of the taproot (JM, Kraft and Pfleger, 2006). Round or irregular light brown lesions that progress to dark black lesions on below-ground stems have also been reported, along with stunting and death (Jung et al., 1999). *Fsp* can survive in the soil for more than one season and conditions that decrease root growth, such as soil compaction, extreme temperatures, and moisture levels, can increase *Fusarium*-mediated root damage (Shaykh et al., 1977; JM, Kraft and Pfleger, 2006).

The development of pea cultivars with root rot resistance has been considered the best long-term management option among the many root rot control strategies (Conner et al., 2014; Bodah et al., 2016; Wang et al., 2018). However, breeding for *Fsp* resistance is challenging, since resistance to *Fsp* is a quantitative trait (Mukankusi et al., 2011; Román-Avilés et al., 2011; Bodah et al., 2016). Furthermore, routine screening for resistance has proven to be time-consuming, expensive, and highly influenced by the environment (Bodah et al., 2016). Marker-assisted selection (MAS) can help expedite the selection of putative *Fsp* resistant progeny without the need for expensive phenotyping. Several efforts have been made to develop molecular markers associated with resistance to *Fsp* root rot in pea (Feng et al., 2011; Coyne et al., 2015, 2019). However, these studies used a limited number of DNA markers and some of the QTLs identified require further fine mapping to provide informative markers due to large confidence intervals (16.8–28.5 cM).

In a preceding time-course transcriptome study, we utilized a combined genetic and RNAseq approach to identify *Fsp*-responsive differentially expressed genes among four partially resistant and four susceptible genotypes (Williamson-Benavides et al., 2020). These genotypes were selected for their contrasting root severity index phenotype (Bodah et al., 2016). Genes involved in secretion and exocytosis, anthocyanin biosynthesis pathway genes, and a previously-described pathogenesis-related (PR) gene DRR230 were observed to be overexpressed in partially resistant genotypes (Chiang and Hadwiger, 1991; Hadwiger, 2008, 2015; Williamson-Benavides et al., 2020). Since the use of single nucleotide polymorphisms (SNPs) can help to refine genetic mapping studies due to their high abundance in the genome (Deulvot et al., 2010), SNPs mined from differentially expressed genes (DEGs) were utilized to identify QTLs associated with *Fsp* resistance using composite interval mapping in two recombinant inbred line (RIL) populations segregating for root rot resistance as observed in greenhouse evaluations.

## Materials and Methods

### Plant Material

The parental plant material used in this study was the same as described in a preceding study (Williamson-Benavides et al., 2020). Briefly, four genotypes with partial resistance to *Fsp*—00-5001, 00-5003, 00-5004, and 00-5007— and four susceptible genotypes— “Aragorn,” “Banner,” “Bolero,” and “DSP”—were selected based on their disease resistance to *Fsp* (Table 1). These genotypes were previously classified as either partially resistant or susceptible based on phenotyping root disease severity index (RDS), plant height, shoot dry weight, and root dry weight after *Fsp* challenge (Bodah et al., 2016). The 5,000 series pea breeding lines were found to be the most resistant lines among the white-flowered pea lines. The susceptible genotypes are among the most frequently used commercial pea varieties in the United States (Table 1).

**TABLE 1:** Selected green-seeded pea genotypes for SNP genotyping [Adapted from Williamson-Benavides et al. (2020)].

Genotype	Source^a^	*Fsp* resistance level^b^	Other disease resistance^c^	100 seed weight (g)	Leaf type^d^	Market class
00-5001	USDA-ARS VFCRU	*	Fop races 1, 2, and 5	22.7	af	Green fresh
00-5003	USDA-ARS VFCRU	*	Fop races 1, 2, and 5	15.9	af	Green fresh
00-5004	USDA-ARS VFCRU	*	Fop races 1, 2, and 5	20.8	af	Green fresh
00-5007	USDA-ARS VFCRU	*	Fop races 1, 2, and 5	22.2	N	Green fresh
“Aragorn”	ProGene	***	Fop races 1, 2; PSBMV	19.5	af	Green dry
“Banner”	ProGene	***	Fop race 2, PM	18.7	af	Green dry
“Bolero”	AsGrow	****	Fop race 1, PM, Pythium, EMV	20.1	N	Green fresh
“DSP”	Canner Seed	***	-	20.9	N	Green fresh

*^*a*^AsGrow, AsGrow Seed Co., San Juan Bautista, CA, United States; Canner Seed, Canner Seed Co., Idaho Falls, ID, United States; ProGene, ProGene LLC Plant Research, Othello, WA, United States; USDA-ARS VFCRU, USDA-ARS, Vegetable and Forage Crops Research Unit, Prosser, WA, United States.*

^*b*^*Fsp* resistance, *Fsp* resistance resulted from phenotyping, from most resistance (*) to susceptible (****) to most susceptible (****) (Bodah et al., 2016).

*^*c*^ Fop, *Fusarium oxysporum*; PSBMV, Pea Seed-Borne Mosaic Virus, PM, Powdery Mildew; EMV, Enation Mosaic Virus.*

*^*d*^af (Afila), semi-leafless; N (Normal), normal leaf type. The table shows seed source, *Fsp* tolerance level, other disease resistance, 100 seed weight, leaf type, and market class per pea genotype.*

The 00-5001, 00-5003, 00-5004, and 00-5007 pea breeding lines were developed by Porter et al. (2014) *via* single-seed descent at USDA–ARS, Prosser, WA. The parentage of 00-5001 is PH14-119/M7477// Coquette/3/86-2197/74-410-2 (Kraft, 1989; USDA–ARS NGRP, 2020). The parentage of 00-5003 is 69PH42-691004/Recette//Popet/3/PH14-119/DL-1/3/B563-429-2/PI257593//DSP TAC (USDA–ARS NGRP, 2020). The parentage of 00-5004 is 79-2022/ICI 1203-1//Menlo/3/PI189171/DL-2//75-786 (Kraft and Tuck, 1986; USDA–ARS NGRP, 2020). The parentage of 00-5007 is 00-5005/00-5006.00-5005 parentage is B669-87-0/M7477//Blixt B5119/3/00-5001/74SN5/3/PH14-119/DL-1//74SN3/Recette/5/FR-725 (Kraft and Giles, 1976; USDA–ARS NGRP, 2020). The parentage of 00-5006 is 00-5003/00-5004.

Two F_7_-derived recombinant inbred line (RIL) populations with 190 individuals each derived from the crosses “Aragorn” × 00-5001 (Population I) and “Banner” × 00-5007 (Population II) were developed by single-seed descent and maintained at ProGene LLC Plant Research, Othello, WA, United States.

### Disease Challenge and Greenhouse Evaluations of Disease

For the two populations, a total of 190 individuals with four replicates each were challenged with three *Fsp* isolates: Fs 02, Fs 07, and Fs 09. These isolates were obtained from infected pea roots collected in the Palouse Region of Washington and Idaho by Dr. Lyndon Porter, USDA-ARS Vegetable and Forage Crops Research Unit, Prosser, WA (United States). The three isolates were single-spored and were identified based on the partial translation elongation factor 1-alpha sequences (Geiser et al., 2004). The pathogenicity of each *Fsp* isolate to pea was also confirmed (Bodah et al., 2016). The three isolates were grown on pentachloronitrobenzene (PCNB) selective media for six days (Nash and Snyder, 1962). Cultures were transferred to KERR’s media (Kerr, 1963) and incubated on a shaker at 120 rpm under continuous light for six days at 23 to 25°C. The spore concentration of each isolate was determined with a hemocytometer and diluted to 1 × 10^6^ spores/ml of water. A spore suspension inoculum was created with equal parts by volume from each of the three isolates.

RIL seeds were surface sterilized in a 0.6% sodium hypochlorite solution and rinsed in sterile distilled H_2_O. The seeds were then soaked for 16 h in the *Fsp* spore suspension as described previously (Bodah et al., 2016). After the challenge with the spore suspension, seeds were planted in a completely randomized design in plastic planter cones (Conetainer, 0.25 L volume, Stuewe and Sons Inc.) filled with a standard perlite medium in a greenhouse at Crites Seed Inc. (Moscow, ID). Plants were irrigated as needed, generally every 24–36 h, and the perlite was watered to saturation at 100% field capacity. A 12-h photoperiod was maintained using 400-watt metal halide lamps for supplemental light. Plants were grown at temperatures ranging between 21–27°C during the day and 12–18°C at night.

Quantitative evaluation of RDS and plant height were recorded 21 days after planting. RDS was evaluated on a visual scale from 0 to 6, in which 0 = no diseases symptoms; 1 = small hypocotyl lesions; 2 = lesions coalescing around epicotyls and hypocotyls; 3 = lesions starting to spread into the root system with some root tips infected; 4 = epicotyl, hypocotyl and root system almost completely infected and limited white, uninfected tissue visible; 5 = completely infected root; and 6 = plant failed to emerge (Bodah et al., 2016). Plant height is a reliable indication of resistance to *Fsp* and height showed the highest negative correlation among all growth parameters related to RDS (Bodah et al., 2016). RDS and height data across the four replicates were averaged for each RIL for further analyses. Phenotyping was repeated twice. Infected root tissue from three inoculated plants was taken at random to verify the presence of *Fsp* in infected tissue. The root tissue was surface sterilized and plated onto PCNB. Culture morphology and growth were observed under a microscope and compared with the original cultures to verify the presence of *Fsp* in the infected tissue.

Broad-sense heritability was estimated with this equation Va/(Va + Ve), where Va represented the genetic variance, Ve the environmental variance.

### DNA Extraction

Leaf tissue was freeze-dried in a lyophilizer. Leaf tissue samples included the eight white-flowered parental genotypes —00-5001, 00-5003, 00-5004, and 00-5007 “Aragorn,” “Banner,” “Bolero,” and “DSP,” as well as the 380 RILs from Population I and Population II. DNA was extracted with the BioSprint 96 DNA Plant kit (Qiagen, Mainz, Germany). A Nanodrop ND-8000 Spectrophotometer (ThermoFisher, MA, United States) was used to quantify the extracted DNA.

### SNP Mining in DEGs, SNP Validation, and RIL Genotyping

A time-course RNAseq analysis, performed on sets of partially resistant and susceptible genotypes after *Fsp* challenge resulted in the identification of 42,905 differentially expressed contigs (DECs) (Williamson-Benavides et al., 2020). SeqMan Pro (DNASTAR, WI, United States) and custom scripts were utilized to identify single nucleotide polymorphisms (SNPs) within the set of 42,905 DECs. The Assay Design Suite software (Agena Bioscience, CA, United States) and the SNP report generated by SeqMan Pro were used to generate two sets of primers for amplifying SNP containing regions (Supplementary Table 1). The high-throughput MassARRAY Technology was used to validate the SNPs. Genotype calling was done from the samples deposited on the chips with the MassARRAY RT v 3.0.0.4 software (Agena Bioscience, CA, United States). Results were analyzed with the MassARRAY Typer v 3.4 software (Agena Bioscience, CA, United States). SNPs were validated across eight pea genotypes “Aragorn,” “Banner,” “DSP,” “Bolero,” 00-5001, 00-5003, 00-5004, 00-5007, which included the four parents of the two segregating populations. Each SNP was screened twice for each individual. SNPs confirmed to be polymorphic between “Aragorn” × 00-5001, and “Banner” × 00-5007 were used -for genotyping of 190 RILs each from Population I and Population II.

### Physical Map and QTL Detection

The physical location of the SNPs used in this study was determined using the pea genome (Kreplak et al., 2019). The SNP marker sequence was aligned *via* BLAST against the complete *P. sativum* genome in URGI BLAST^1^. RDS and height averages for each RIL were used to map the QTLs associated with resistance to *Fsp*. QTLs were detected with the composite interval mapping (CIM) function of the R statistical software version 3.0.2 (R core team, Vienna, Austria). CIM default settings were used. The Kosambi map function was applied to impute missing marker genotype data. QTLs were considered significant above the threshold LOD score 3.0. QTLs were named with the prefix *WB.Fsp-Ps* followed by the chromosome number and the QTL number within the chromosome.

### Functional Annotation of QTLs Associated With Fsp Resistance in Pea

The QTLs *WB.Fsp-Ps* 5.1, *WB.Fsp-Ps* 5.2, *WB.Fsp-Ps* 5.3, *WB.Fsp-Ps* 2.1, and *WB.Fsp-Ps* 3.1 were annotated using the functional annotation and gene ontology (GO) data generated in a preceding study (Williamson-Benavides et al., 2020). QTL annotation provided the identity of genes and DECs located within the selected genomic regions. The confidence intervals for each of the QTLs were taken into account to identify the genomic sequence of each QTL from the pea genome (Kreplak et al., 2019). The transcriptome data were aligned *via* BLAST against the QTL sequence regions in CLC Bio Genomics Workbench 6.0.1 (CLC Bio, Aarhus, Denmark).

## Results

### Disease Challenge and Greenhouse Phenotypic Evaluation

**FIGURE 1:**
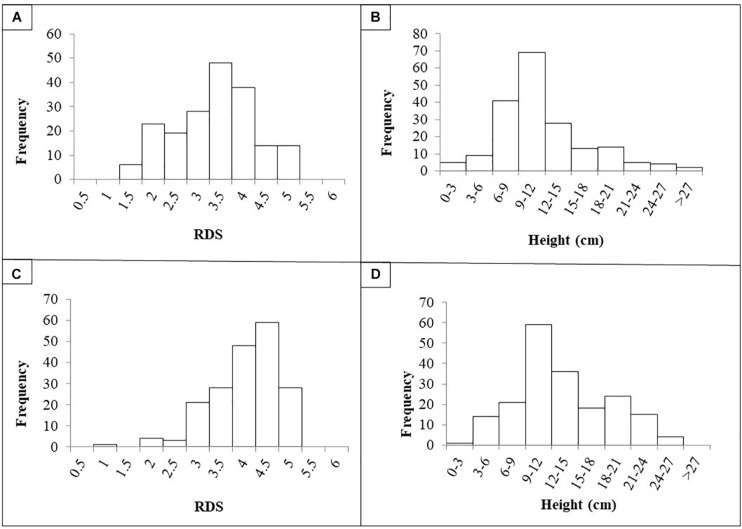
Frequency histograms of root disease severity (RDS) and plant height of recombinant lines (RILs) after challenge with *Fusarium solani* f. sp. *pisi.* RILs were derived from crosses “Aragorn” × 00-5001 **(A,B)** and “Banner” × 00-5007 **(C,D)**.

The quantitative evaluation of RDS and plant height was averaged per RIL across the four replicates as there was no significant difference between the replicates (*p* < 0.05). Frequency histograms for both traits per population are presented in Figure 1. The phenotypic means for the parents for Population I were Aragorn-RDS = 4.5; Aragorn-Height = 7.0; 00-5001-RDS = 2.5; and 00-5001-Height = 10.5. The phenotypic means for the parents for Population II were Banner-RDS = 3.3; Banner-Height = 13.0; 00-5007-RDS = 2.3; and 00-5007-Height = 10.0. The two RIL populations displayed transgressive segregation for both increased susceptibility and resistance over the two parental lines as measured by RDS and height traits (Figure 1).

Based on the Shapiro–Wilk test, in Population I, data were not normally distributed for RDS [*W*(189) = 0.95, *p* < 0.01] or for height [*W*(189) = 0.91, *p* < 0.01]. Similarly in Population II, data were not normally distributed for RDS [*W*(189) = 0.90, *p* < 0.01] or for height [*W*(189) = 0.97, *p* < 0.01]. A significant negative correlation was found between the RDS and height values for Population I [*r*(188) = −2.76, *p* < 0.01] and Population II [*r*(188) = −4.38, *p* < 0.01]. For Population I, broad sense heritability was 49.8 and 70.5% for RDS and height, respectively. For Population II, broad sense heritability was 43.1 and 83.4% for RDS and height, respectively.

### SNP Screening, SNP Validation, and RIL Genotyping

**FIGURE 2:**
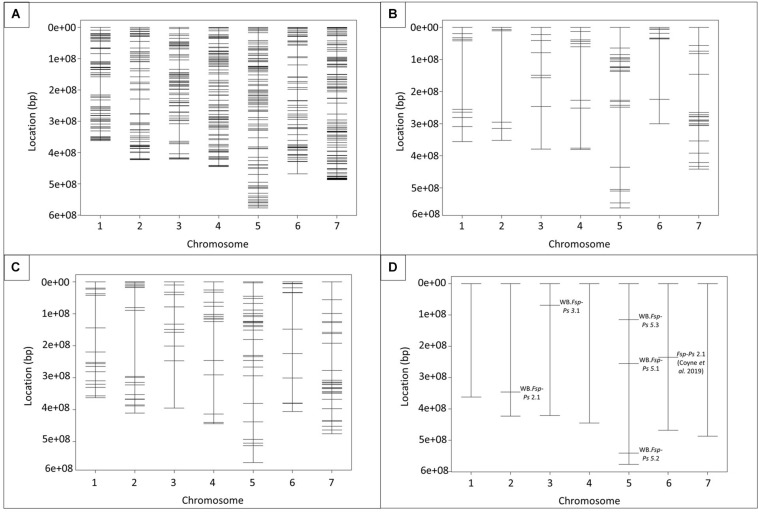
Physical maps of SNPs and QTLs identified in the seven *Pisum sativum* chromosomes. Location of 769 SNPs, mined from *Fsp*-responsive differentially expressed genes (DEGs), in pea genome **(A)**. Total of 100 and 154 SNPs with validated polymorphisms for population I (“Aragorn” × 00-5001) **(B)** and population II (“Banner” × 00-5007) **(C)**, respectively. Five QTLs were identified in association with disease resistance **(D)**. Major QTL *Fsp-Ps* 2.1 (Coyne et al., 2019) was added as a reference **(D)**. Physical distances, represented in base pairs (bp), are shown on the left side of the graphs.

SeqMan Pro (DNASTAR, WI, United States) identified a total of 769 SNPs across DECs (Figure 2A and Supplementary Table 2). The predicted SNPs were validated in the “Aragorn,” “Banner,” “DSP,” “Bolero,” 00-5001, 00-5003, 00-5004, and 00-5007 pea genotypes (Supplementary Table 3). A total of 118 SNPs were confirmed for cultivars DSP and Bolero while 256 SNPs were confirmed for 5007 and Banner (Table 2). SNPs confirmed to be polymorphic between “Aragorn” × 00-5001 (219 SNPs) (Figure 2B) and “Banner” × 00-5007 (256 SNPs) (Figure 2C) were used to screen 190 individuals each of RIL Populations I and II, respectively. The screening results of 190 individuals each for both Population I and II are summarized in Supplementary Tables 4, 5, respectively.

**TABLE 2:** Number of SNPs across eight *Pisum sativum* genotypes from a total of 769 predicted SNPs.

Genotype	Aragorn	Banner	Bolero	DSP	5001	5003	5004	5007
Aragorn	-	162	234	229	219	225	214	227
Banner	162	-	224	239	252	219	231	256
Bolero	234	224	-	118	191	220	191	213
DSP	229	239	118	-	206	223	177	218
5001	219	252	191	206	-	157	166	149
5003	225	219	220	223	157	-	200	181
5004	214	231	191	177	166	200	-	208
5007	227	256	213	218	149	181	208	-

### Physical Map and QTL Detection

The physical genomic location of all the SNPs used in this study was determined using the pea genome (Kreplak et al., 2019; Figure 2 and Supplementary Table 6). Chromosome 1, 2, 3, 4, 5, 6, and 7 registered a total of 92, 86, 84, 122, 118, 78, 139 SNPs, respectively (Figure 2 and Supplementary Table 6). A total of 47 SNP markers were identified in 42 scaffolds that had not been assigned to any of the seven chromosomes of pea (Supplementary Table 6). Three of the predicted SNP markers were not localized on the pea genome.

Prior to QTL mapping for Population I and II, a quality assessment of the genotypic data was performed. Individuals and markers with more than 80% of missing data were omitted in each database. Markers with distorted segregation patterns were also removed from the data for QTL analysis. A total of 190 RILs and 100 markers were used for QTL analysis of Population I (Figure 2B and Supplementary Table 7). A total of 182 individuals and 154 markers were used for QTL analysis of Population II (Figure 2C and Supplementary Table 8). Means per RIL for RDS and height were used to map the QTLs associated with resistance to *Fsp*. Five different QTLs were identified in the two RIL populations for RDS and height (Figure 2D and Table 3). These QTLs explained 5.3 to 14.8% of the phenotypic variance (Table 3).

**TABLE 3:** Quantitative trait loci detected for resistance to *Fusarium solani* f. sp. *pisi* root rot in two RIL populations using root disease severity (RDS) and height.

Population	Scoring phenotype	QTL name	Chromosome	Position	Marker	LOD peak	LOD-1.5 support interval (cM)	LOD-1.5 support interval (nt)	*R* ^2^
Population I	RDS	*WB.Fsp-Ps* 5.1	5	42.9	1_14714	3.7	40.8–44.0	236,573,488–254,762,537	8.3
	Height	*WB.Fsp-Ps* 5.2	5	95.4	7401	4.4	89.1–98.2	515,954,961–569,073,935	7.5
Population II	RDS	*WB.Fsp-Ps* 3.1	3	9.9	26703	3.4	6.0–17.9	34,871,998–103,747,090	8.1
		*WB.Fsp-Ps* 5.3	5	19.2	3_31807	3.1	17.6–22.6	101,835,502–131,088,590	5.3
	Height	*WB.Fsp-Ps* 5.1	5	42.9	1_14714	6.4	40.8–51.2	236,515,561–296,527,837	14.8
		*WB.Fsp-Ps* 2.1	2	58.2	20326	3.2	57.0–63.4	330,067,516–367,256,590	5.6

*The RIL populations were derived from the following crosses: “Aragorn” × 00-5001 (Population I) and “Banner” × 00-5007 (Population II).*

### Functional Annotation of QTLs Associated With Fsp Resistance in Pea

The transcriptome data, generated previously (Williamson-Benavides et al., 2020) were aligned *via* BLAST with the 5 QTLs: *WB-Fsp-Ps* 5.1 (Supplementary Table 9), *WB-Fsp-Ps* 5.2 (Supplementary Table 10), *WB-Fsp-Ps* 5.3 (Supplementary Table 11), *WB-Fsp-Ps* 2.1 (Supplementary Table 12), and *WB-Fsp-Ps* 3.1 (Supplementary Table 13). From the total set of aligned genes, 119–133 genes per QTL had previously been classified as differentially expressed (Supplementary Tables 9–13). A total of 3–17 DEGs and 6–11 non-DEGs were also identified, predicted as having unknown function or annotated as hypothetical proteins in QTLs *WB-Fsp-Ps* 5.1, 5.2, 5.3, 2.1, and 3.1 (Supplementary Tables 9–13).

A total of 7 DEGs associated with disease response were found in *WB-Fsp-Ps* 5.1. These genes are involved in the synthesis of lipids (acetyl-CoA carboxylase)**;** cell signaling (C-type lectin receptor-like tyrosine-protein kinase and MAPK)**;** nodulation (nodulation-signaling pathway 2 protein), and protein degradation (F-box/kelch-repeat). Another ten genes in *WB-Fsp-Ps* 5.1 were associated with disease resistance; however, these genes did not exhibit differential expression (Williamson-Benavides et al., 2020). These set of genes is associated with synthesis of lipids (1 gene)**;** auxin signaling (2), ethylene synthesis (1), pectin synthesis (1), and regulation of transcription (5).

Seventeen genes found in *WB-Fsp-Ps* 5.2 were associated with disease response and also showed differential expression after *Fsp* challenge. This list included three PR (pathogenesis-related) genes (universal stress protein PHOS32-like, endochitinase PR4, protein enhanced disease resistance 2); an anthocyanin 5-aromatic acyltransferase; four receptor-like kinases; and seven TFs of the GATA, NLP8, C2H2, and scarecrow types. Another set of seventeen contigs were identified as candidate genes but did not show any differential expression. The genes on the latter list are associated with synthesis of lipids (ketoacyl-CoA synthase); three transcription factors (GLABRA and PosF21 type); an endochitinase PR4**;** four receptor like-kinases; and two universal stress proteins PHOS32.

Twenty-two DEGs in *WB-Fsp-Ps* 5.3 were associated with disease response mechanism. This list included drug transporters (ABC transporters); a cluster of seven F-box proteins; genes involved in cell wall biosynthesis and modification (pectinesterase/pectinesterase inhibitor and polygalacturonase); the TFIIS TF; and two PR proteins—protein enhanced disease resistance 4-like and pathogenic type III effector avirulence factor. Three more ABC transporters were found in the *WB-Fsp-Ps* 5.3, but they did not show any differential expression. Other candidate genes found in *WB-Fsp-Ps* 5.3 that did not show differential expression included an autophagy-related protein; a brassinosteroid receptor; another F-box gene; five more receptor kinases; a protein enhanced disease resistance 4-like and pathogenic type III effector avirulence factor; two TFs [CCHC(Zn) family and ERF110]; and a UDP-glucuronate:xylan alpha-glucuronosyltransferase 1.

Fourteen DEGs involved in disease resistance mechanism were associated with *WB-Fsp-Ps* 2.1. These genes are known to participate in cell membrane synthesis and modification (CSC1 protein and sphingolipid transporter)**;** PR gene response (disease resistance protein RPM1 and disease resistance protein RGA3); regulation of transcription (Myb/SANT and ninja-family protein AFP3); and cell signaling (receptor-like protein kinase 2). Three genes involved in cell wall and membrane synthesis/modification (glycerol-3-phosphate acyltransferase, sphingolipid transporter, and 3UDP-arabinopyranose mutase); and nine TFs (Myb/SANT, ninja-family protein AFP3, PLATZ transcription factor family protein) were also identified as potential candidates that contribute to the effect of *WB-Fsp-Ps* 2.1. However, this set of genes did not show differential expression after *Fsp* challenge.

Eleven DEGs associated with disease response were found within *WB-Fsp-Ps* 3.1. These genes were annotated as ethylene response sensors; polygalacturonase inhibitors; phopholipases; receptor kinases; and NDR1/HIN1-like protein 10. A set of fourteen genes were characterized as associated with disease response, however they did not show differential expression. This list contains cathepsin B-like protease 2; ethylene-insensitive protein 2; F-box protein PP2-A15 isoform X2; mannan synthase 1-like isoform X1; polygalacturonase inhibitor; nuclear transcription factor Y subunit B-10; and serine/threonine protein receptor genes.

## Discussion

Here, we have reported the identification of five QTLs that are associated with *Fsp* resistance in pea (Figure 2D and Table 3). Each of these QTLs explains 5.3–14.8% of the total phenotypic variation, and together they add up to 33.21% of the variation. These five QTLs were identified by using polymorphisms embedded in *Fsp*-responsive DEGs. These polymorphisms and DEGs were originally identified *via* RNAseq. The identification of DEGs that respond to or are associated with specific biotic or abiotic stimulus, as well as the development of markers embedded in these DEGs is an efficient alternative to genotyping by sequencing for fine mapping.

To the best of our knowledge, this is the only study to report the presence of QTLs associated with *Fsp* resistance on chromosomes 2 and 3 of pea (Figure 2D and Table 3). A total of three QTLs were identified on chromosome 5. Previously, three QTLs associated with *Fsp* resistance, *Fsp-Ps 3*.1, 3.2 and *3*.3, had been reported on chromosome 5 (Coyne et al., 2015, 2019). QTLs *Fsp-Ps 3*.2 and *Fsp-Ps 3*.3 are located close to each other and adjacent to the newly identified *WB-Fsp-Ps* 5.3. Further fine mapping should be able to determine if these three QTLs are in fact three, two, or only one QTL. The situation is similar in the case of QTLs *WB-Fsp-Ps* 5.1 (Figure 2D and Table 3) and *Fsp-Ps 3*.1 (Coyne et al., 2015). LOD intervals from these two QTLs do not overlap either, although they are located close to one another on chromosome 5. This proximity can mean that they represent one QTL.

Interestingly, this study did not find any QTLs on chromosome 6. *Fsp-Ps* 2.1 (Coyne et al., 2019; Figure 2B) and a QTL identified by Feng et al. (2011) located on chromosome 6 explained 44.4–53.4 and 39% of the phenotypic variance, respectively. These two remain the major QTLs identified so far for *Fsp* resistance in pea. The absence of a major QTL on chromosome 6 in this study could be due to the diversity of the parental source of resistance used in this study (005001 and 00-5007), versus what was used in previous studies (PI 180693, PI 557501, “Carman”) (Feng et al., 2011; Coyne et al., 2015, 2019). The large effect shown by *Fsp-Ps* 2.1, seen in previous reports, may explain the bimodal distribution for traits such as the root severity index, plant height, and plant weight (Coyne et al., 2019). However, data presented in this study did not show a bimodal distribution but showed a trend toward normal distribution, which might explain the absence of QTLs with large effects.

The establishment of associations between disease-related genes and resistance, or susceptibility can facilitate the understanding of the possible mechanism(s) involved in the pathogenicity of *Fsp* in pea. *WB-Fsp-Ps* 5.1 was the major QTL identified in this study. Among the DEGs identified in this QTL, an F-box/kelch-repeat protein (DN2516_c0_g1_i1) demonstrated reduced expression at 12 h (FC = −24.4) after *Fsp* challenge in the partially resistant, but not in the susceptible genotypes. Nine F-box protein–coding genes have been found in the region of a highly dominant QTL that provides resistance to *A. euteiches*, a root rot pathogen in pea (Djébali et al., 2009; Pilet-Nayel et al., 2009). F-box proteins are known to be involved in hormone regulation and in plant immunity (Guo and Ecker, 2003; Lechner et al., 2006). A nodulation signaling gene found in *WB-Fsp-Ps* 5.1 was also found to be upregulated at 0 h (FC = 2.4) in susceptible genotypes when the expression levels were compared against the expression levels in partially resistant genotypes. It has been reported that a central regulator of symbiotic nodule development is determinant of susceptibility toward *A. euteiches* in *Medicago truncatula* (Rey et al., 2013).

Several genes associated with disease resistance were located in *WB-Fsp-Ps* 5.2 QTL region (Supplementary Table 10). Contigs DN19556_c0_g1_i1, DN2007_c0_g1_i4, DN77_c0_g1_i6 were identified as anthocyanin 5-aromatic acyltransferase, endochitinase PR4, and protein enhanced disease resistance 2. These genes showed differential expression; their expression was significantly higher in the susceptible genotypes compared to the partially resistant genotypes (Williamson-Benavides et al., 2020). The same pattern was observed for membrane receptors in all five QTLs; TFs present in *WB-Fsp-Ps 5.2*; NDR1/HIN1-like protein 10 and polygalacturonase inhibitors found in *WB-Fsp-Ps* 3.1; the RPM1-like and putative disease resistance protein RGA3 identified in *WB-Fsp-Ps* 2.1; as well as for a cluster of F-box proteins, a pathogenic type III effector avirulence factor, a pectinesterase/pectinesterase inhibitor, and a protein enhanced disease resistance 4-like gene found in *WB-Fsp-Ps* 5.3 (Supplementary Tables 9, 10,12). The high expression of any of these genes might be associated with disease susceptibility. However, further reverse genetics analyses will need to be performed to determine the dominant or recessive nature of these genes and QTL(s).

Cell death in the pea-*Fsp* interaction can help in the progression of *Fsp* infection due to the necrotrophic nature of the *Fsp* pathogen (Williamson-Benavides et al., 2020). The contig DN352_c0_g1_i17 located within *WB-Fsp-Ps* 5.3 was identified as CPR-5 protein which is known to negatively regulate the senescence and chlorotic lesions induced by pathogens when controlling programmed cell death (Bowling et al., 1997; Yoshida et al., 2002). This gene is highly suppressed in expression at 12 h (FC = −3.03) after *Fsp* challenge in the susceptible genotype, which might trigger cell death.

Gene DN813_c0_g1_i4, located in *WB-Fsp-Ps* 2.1, was highly overexpressed in the partially resistant genotype when compared against the expression values in the susceptible genotypes under controlled conditions at 6 (FC = −4.76) and 12 h (FC = −2.71) and under *Fsp*-inoculation at 0 (FC = −3.76) and 12 h (FC = −2.29). BLAST search of contig TRINITY_DN813_c0_g1_i4 identified it as *Medicago truncatula* 1-aminocyclopropane-1-carboxylate oxidase homolog 1 (XM_003627980) (e-value: 5e-165, percentage identity: 84.5%). The 1-aminocyclopropane-1-carboxylate oxidase enzyme is involved in the production of ethylene. Jasmonate-induced defense responses, the expected response to counter the presence of necrotrophic pathogens such as *Fsp*, are known to be associated with elevation of 1-aminocyclopropane-1-carboxylate oxidase and also to increase the activity of defense-related enzymes and subsequent control of disease incidence (Yu et al., 2011; Dixit et al., 2016). Another R gene located in *WB-Fsp-Ps* 2.1 is the putative disease resistance protein RGA3; however, differential expression was not observed for this gene (Williamson-Benavides et al., 2020).

We identified transgressive segregation in the two populations in study. Several of the transgressive lines showed enhanced resistance. For instance, a total of 29 and 5 RILs are more resistant than 00-5001 and 00-5007, respectively, based on their RDS scores. Genotypes 00-5001 and 00-5007 were previously characterized as high yielding varieties with important agronomics such as higher resistance to *Fsp*, resistance to Fusarium wilt, semi-leafless leaf type, and anti-lodging characteristics. Therefore, the transgressive lines with enhanced resistance might serve as potential candidate cultivars with good agronomics and even higher resistance to *Fsp*. Yields of these transgressive lines can be compared to elite cultivars under controlled and root rot conditions.

## Conclusion

The use of polymorphic DEGs for QTL mapping resulted in the identification of a new major QTL *WB.Fsp-Ps* 5.1 as well as other four minor QTLs. This outcome indicates that a combined gene expression and genetics approach is effective in identifying genomic regions that may otherwise remain undetected especially for quantitative traits. Chromosome 5 is a source of several QTLs associated with *Fsp* resistance. Some of these QTLs on chromosome 5 are closely located to each other, which is a sign of resistance islands. Clustered resistance genes within the same genetic locus (resistance islands) can be transferred *en bloc* to new pea varieties through breeding. Selection toward these newly identified QTLs, along with previously identified QTLs, should allow for rapid improvement of resistance to *Fsp* root rot in the commercial pea genotypes. Furthermore, candidate genes, nested in each QTL will be instrumental in furthering our understanding of *Fsp*-pea interactions.

## Data Availability Statement

The raw sequencing data used for RNAseq analysis and identification of polymorphic regions is publicly available from the NCBI Sequence Read Archive (SRA, https://www.ncbi.nlm.nih.gov/sra) under the accession number PRJNA630497.

## Author Contributions

AD, EB, RS, and BW-B designed the study. BW-B, GN, and EB performed the experiments and generated the data. BW-B analyzed the data. LP provided the tolerant pea genotypes and *Fsp* isolates. AD supervised the study. All authors read and approved the final manuscript.

## Conflict of Interest

The authors declare that the research was conducted in the absence of any commercial or financial relationships that could be construed as a potential conflict of interest.

## Publisher’s Note

All claims expressed in this article are solely those of the authors and do not necessarily represent those of their affiliated organizations, or those of the publisher, the editors and the reviewers. Any product that may be evaluated in this article, or claim that may be made by its manufacturer, is not guaranteed or endorsed by the publisher.

## Footnotes

1
https://urgi.versailles.inra.fr/blast/
